# U.S.-Imposed Economic Sanctions on Iran in the COVID-19 Crisis From the Human Rights Perspective

**DOI:** 10.1177/00207314211024912

**Published:** 2021-06-09

**Authors:** Abbas Karimi, Hojjat Salimi Turkamani

**Affiliations:** 1 48432 Tabriz University of Medical Sciences, Tabriz, Iran; 2 125619 Azarbaijan Shahid Madani University, Tabriz, Iran

**Keywords:** COVID-19, sanctions, human rights, Iran, US

## Abstract

One of the obvious impacts of comprehensive economic sanctions is on pharmaceuticals and supplies, which are essential elements of all functioning health systems. Observers report that comprehensive sanctions imposed on Iran are a barrier to Iran's coronavirus disease-2019 (COVID-19) crisis and could impede Iran's access to coronavirus vaccines. In this minireview, we discuss COVID-19 and its human rights dimensions of U.S. sanctions on Iran. We believe fighting COVID-19 with limited resources during sanctions will produce a humanitarian crisis, and coronavirus is a convincing reason to lift the sanctions on Iran.

As an alternative to military confrontation, the United States has imposed sanctions over the countries that do not follow U.S. policies. Since 1979, U.S.-imposed sanctions on Iran have been applied.^1^ These sanctions have various effects on trade, banking, investment, and public health. Although the United States says food, medicine, and other humanitarian supplies are exempt from sanctions, it impacts the availability of basic medications and critical raw materials needed for their production.^1,2^ The American Association for World Health has stated that the U.S. embargo of Cuba has caused a significant rise in suffering—and even deaths. Indeed, “economic sanctions are war against the public health system.”^3^

Science, technology, and international research collaboration are drastically affected by economic sanctions. As most research support in Iran is from the government, a decrease in government income will decrease the quality and quantity of research. A decline in health care services under sanctions subsequently leads to limited access to life-saving medicines.^4^

## The COVID-19 Health Crisis

The COVID-19 spread started in late December 2019 in Wuhan, China. After 2 months, this deadly disease spread rapidly throughout the world. On March 12, 2020, the World Health Organization (WHO) announced the COVID-19 pandemic, and the outbreak still constitutes a public health emergency of international concern.^5^ In the first days of the outbreak, the disease encountered most counties with economic and social ramifications, and it continues. European and North American countries could not control the spread as fast as China due to the inability to enforce restrictions.

In Iran, the first confirmed cases were reported on February 19, 2020, in Qom (Qom Province, Iran) and then spread across the country. According to Iran's Ministry of Health, as of April 1, 2021, at least 1,885,564 confirmed COVID-19 cases and 62,665 deaths had been reported.^6^ Epidemic estimation studies for Iran claim that the exact number of daily deaths or cases of COVID-19 is much greater than those in Iran's official reports.^7^ Following COVID-19 restrictions in Iran, the collapse of economic activity and financial markets began. The government entered an economic crisis alongside the U.S.-imposed embargo. This outbreak became the latest point of contention between the United States and Tehran. Despite the international community's and Iran's request for the lifting of U.S. sanctions, they remained intact regarding the United States. The quick spread of disease poses a severe challenge to the health care system and posed barriers to the government in providing humanitarian goods—such as medicine and humanitarian supplies.^8^ Sanctions hindered Iran's access to the international financial system, limiting Iran's ability to buy and ship goods not prohibited by sanctions, and could impede Iran's access to coronavirus vaccines. Here we discuss the human rights dimension of the sanctions’ impact on innocent people who are not parties to this dispute.

## Human Rights Dimensions of COVID-19

Most international human rights documents, including the Universal Declaration of Human Rights of 1948 (UDHR), the International Covenant on Political and Civil Rights of 1966 (ICPCR), and the International Covenant on Economic, Social, and Cultural Rights of 1966 (ICESCR), support the right to health and access to medicine. Article 25 of UDHR refers to a person's right to a standard of living that allows him or her to maintain health and well-being; this includes access to food and medical care.^9^ Also, ICPCR protects individuals or groups from certain forms of oppression.^10^ In 1966, ICESCR proclaimed that all persons had a right to the highest attainable standard of physical and mental health; it called on all involved countries to ensure the prevention, treatment, and control of diseases and to create conditions that would ensure the delivery of medical care (Articles 12.1 and 12.2[c]).^11^ Although the content of the UDHR is not binding on member states, the contents of 2 covenants, as international treaties, are obligatory and binding on all member states. The United States ratified ICPCR in 1992 and as a member state would need to comply with all its provisions. Concerning ICESCR, the United States signed in 1977, but has not yet ratified and is not a party to the treaty. However, under the principles of international law, widespread ratification of international human rights instruments can establish customary international law. In this case, they might become binding on all states regardless of ratification. Due to its widespread ratification internationally (171 states), ICESCR is arguably customary international law and might become binding on the United States, irrespective of its ratification. As a consequence, the United States is arguably committed to complying with the right to health enshrined in Article 12 of ICESCR.^12^

In addition to international treaties and documents on the right to health, several organizations ensure this right in the global aspect. The World Health Organization (WHO) is a well-established and professional organization that directly governs activities of states in the health area. Statutes and decisions of different organs of WHO make obligations on member states. According to the constitution of WHO, as a specialized agency within the terms of Article 57 of the Charter of the United Nations, to achieve its objective, the functions of the WHO shall be “to act as the directing and coordinating authority on international health work” and “to assist states in strengthening health services.” Although the WHO is a practical and institutional body, it represents and specifies some rules and definitions of international norms and terms. For example, the WHO defined health as a “state of complete physical, mental, and social well-being and not merely the absence of disease or infirmity” that is still applied by modern authorities. In this regard, “the promotion and protection of human rights and promotion and protection of health are fundamentally linked” to ensure the advancement of human well-being.^3,13^

According to international treaties, all states have 2 kinds of obligations against other member states: positive and negative. In the context of negative obligations, member states must not make obstacles to other states in fulfilling human rights obligations. In addition, in the context of positive obligations, member states must help other states fulfill and perform their human rights obligations. Hence, the United States, as a member state of international human rights treaties, shall not only notblock access of Iran to financial resources but also must supply Iran medicines and pharmaceuticals or financial help.

It becomes apparent that stifling the economic lifeline of a country through sanctions curtails the development of the economy and the health of individual persons as a respected voice in medicine. So, patients' rights and physicians’ responsibilities will directly be impacted by sanctions. Patients' dissatisfaction and growing uneasiness with unequal access to medical care and the COVID-19 vaccination program in the socioeconomic and political crisis of COVID-19 has created a serious challenge for Iran's government.

The COVID-19 crisis has constrained the economic activity of many countries, especially Iran, that are under U.S. sanctions. Health care and public health professions are having a severe impact from the coronavirus and higher suffering from this crisis. In the early days of the COVID-19 outbreak in Iran, the lack of proper masks, gowns, and eye gear in hospitals was evident, and it persists. Shortages of test kits, protective equipment, ventilators, pharmaceuticals, and supplies, which are essential elements of all functioning health care systems, are directly related to comprehensive economic sanction effects.

The negative consequences of economic sanctions during the COVID-19 crisis are unacceptable from the human rights perspective; they harm the population and effectively limit full access to humanitarian goods, which is a violation of the international human rights obligations of the United States. In the context of Draft Articles on Responsibility of States for Internationally Wrongful Acts, adopted by the International Law Commission at its 53rd session (2001), breach of international obligations by a state can lead to the international responsibility of the state. In addition to other solutions and remedies, in the first step, the responsible state must stop the continuation of the wrongful act and make a promise not to commit it again. In light of this rule, the United States must stop financial sanctions on Iran and not enact new sanctions during the coronavirus pandemic.^14^ The United States has declared that humanitarian goods, such as food- and health-related materials or medical supplies, are exempt from sanctions, saying there is no legal barrier to Iran for procurement of vaccines through the COVAX Facility. Iran's officials say due to the lack of payment mechanism and financial channels for COVAX vaccines, COVID-19-associated morbidity and mortality may have a higher rate among civilians.

## Conclusions

Clearly, the decades-long economic sanctions regime has had a severe, detrimental public health impact, and led to poor health outcomes among Iranians. It is time to take action to lift these oppressive sanctions or to help shape the structure and application of economic sanctions to ensure they protect the health of the persons that are subject to them.
